# Progression patterns and site‐specific responses in advanced gastric cancer patients treated with nivolumab

**DOI:** 10.1002/cam4.5689

**Published:** 2023-02-15

**Authors:** Toru Kadono, Satoru Iwasa, Kengo Nagashima, Kotoe Oshima, Shun Yamamoto, Hidekazu Hirano, Natsuko Okita, Hirokazu Shoji, Yoshitaka Honma, Atsuo Takashima, Ken Kato, Toshikazu Ushijima, Narikazu Boku

**Affiliations:** ^1^ Department of Gastrointestinal Medical Oncology National Cancer Center Hospital Tokyo Japan; ^2^ Biostatistics Unit, Clinical and Translational Research Center Keio University Hospital Tokyo Japan; ^3^ Department of Head and Neck, Esophageal Medical Oncology National Cancer Center Hospital Tokyo Japan; ^4^ Division of Epigenomics National Cancer Center Research Institute Tokyo Japan

**Keywords:** gastric cancer, immune checkpoint inhibitors, multivariate analysis, nivolumab, prognosis

## Abstract

**Background:**

While the efficacy of immune checkpoint inhibitors (ICIs) reportedly varies among metastatic sites and progression patterns (classified as systemic progression [SP] or mixed progression [MP]), the clinical efficacy of ICIs against gastric cancer remains unclear. The response to nivolumab depending on metastatic site and clinical outcomes according to progression pattern in patients with advanced gastric cancer was investigated retrospectively.

**Methods:**

Seventy‐four advanced gastric cancer patients with measurable lesions who received nivolumab monotherapy between 2015 and 2020 were enrolled. Progression‐free survival (PFS), overall survival, response at each metastatic site, and clinical outcomes according to progression pattern were analyzed retrospectively. SP and MP were defined as progression in more than half of the lesions and progression in half or fewer of the lesions, respectively, in cases evaluated as progressive disease.

**Results:**

Thirty‐five (47%) and 27 (36%) patients had SP and MP, respectively, and 12 (16%) patients experienced no progression. The progression rates of target lesions in the lung (44%) and liver (57%) were significantly higher than that in the lymph nodes (18%) (lung vs. lymph node, *p* < 0.001; liver vs. lymph node, *p* = 0.03). Patients with MP had superior PFS to those with SP (median, 2.6 vs. 1.5 months; HR, 0.42; 95% CI, 0.23–0.76; *p* = 0.004). In MP group, patients with treatment beyond progression (TBP) with nivolumab had a trend of longer post‐progression survival than those without TBP (median, 8.0 vs. 4.0 months; HR, 0.55; 95% CI, 0.23–1.29; *p* = 0.161).

**Conclusion:**

Patients with MP had a longer PFS than those with SP. Lung and liver metastases had a poorer response to an ICI than lymph node metastases.

## Graphical abstract

In advanced gastric cancer treated with nivolumab, patients with mixed progression had a longer progression‐free survival than those with systemic progression. Lung and liver metastases had a poorer response to nivolumab than lymph node metastases.

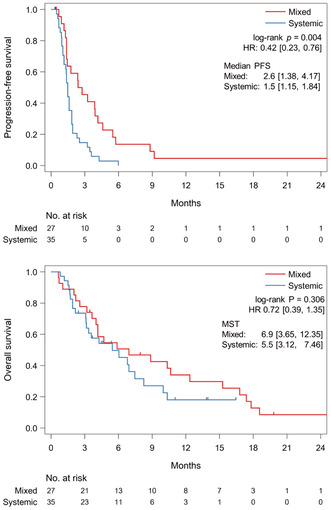

## 1 INTRODUCTION

Immune checkpoint inhibitors (ICIs), such as programmed death‐1 (PD‐1) inhibitors, have shown better clinical outcomes of patients with various malignancies.^1^, ^2^, ^3^, ^4^, ^5^, ^6^, ^7^ The interaction between PD‐1 expressed on the surface of T‐cells and programmed death‐ligand 1 (PD‐L1) on cancer cells and immune cells downregulate T‐cell activation.^8^, ^9^ Therefore, blocking the PD‐1/PD‐L1 pathway has anti‐tumor effects. Nivolumab is a humanized monoclonal IgG4 PD‐1 antibody that has been approved as a third‐ or later‐line treatment for unresectable advanced or recurrent gastric cancer based on the phase III ATTRACTION‐2 trial,^10^, ^11^, ^12^, ^13^ in which the median overall survival (OS) was better in the nivolumab group than in a placebo group (median, 5.26 vs. 4.14 months; HR, 0.63; 95% CI, 0.51–0.78; *p* < 0.0001). Subsequently, nivolumab combined with chemotherapy as a first‐line treatment has shown favorable survival in the CheckMate 649 trial and the ATTRACTION‐4 trial^14^, ^15^; however, the prognosis of patients with advanced gastric cancer remains poor.

Recently, some studies have shown different responses to ICIs according to metastatic site,^16^, ^17^, ^18^, ^19^, ^20^, ^21^, ^22^ and the presence of liver metastasis was reportedly related to a significantly lower objective response rate (ORR) in patients with melanoma, NSCLC, and urothelial carcinoma.^16^, ^17^, ^18^, ^19^, ^20^, ^21^ In addition, Osorio et al.^16^ discussed that spatiotemporal pattern of response to ICIs in individual metastases tend to be homogeneous, whereas progression patterns were heterogeneous. They defined “systemic progression (SP)” as progressive disease (PD) in more than one lesion (including target, nontarget, and new lesions) in patients with fewer than four target lesions or PD in more than two lesions (including target, nontarget, or new lesions) in patients with more than three target lesions; in contrast, “mixed progression (MP)” was defined as PD in only one lesion (including target, nontarget, and new lesions) in patients with fewer than four target lesions or PD in fewer than three lesions (including target, nontarget, and new lesions) in patients with more than three target lesions. NSCLC patients with MP showed better PFS and OS than patients with SP.^16^ The data on the response to nivolumab depending on the metastatic site and the relation between progression patterns and prognosis in patients with advanced gastric cancer is limited.

Furthermore, in patients with advanced gastric cancer, a post‐hoc analysis of the ATTRACTION‐2 trial suggested that treatment beyond progression (TBP) with nivolumab might confer a better post‐progression survival (PPS) than a placebo, and the progression patterns were associated with the survival of TBP with nivolumab.^13^ However, whether the progression patterns are correlated with the prognosis of patients treated with nivolumab and whether nivolumab should be continued beyond progression depending on the progression pattern remain unclear.

In this retrospective study, we investigated the response to nivolumab depending on metastatic site and clinical outcomes according to progression pattern in patients with advanced gastric cancer. Additionally, whether the benefit of TBP with nivolumab depends on progression patterns were also investigated.

## 2 METHODS

### 2.1 Patients

We retrospectively analyzed consecutive advanced gastric cancer patients who received nivolumab monotherapy as a third‐ or later‐line treatment between March 2015 and May 2020 at the National Cancer Center Hospital, Tokyo, Japan. Both doses of nivolumab, 3 mg/kg and 240 mg/body every 2 weeks, were acceptable to the subject. The main selection criteria for this retrospective study were as follows: (a) gastric or esophagogastric junction adenocarcinoma in histology; (b) prior treatment with two or more lines of chemotherapy, including fluoropyrimidine‐ and taxane‐based regimens; (c) an Eastern Cooperative Oncology Group Performance Status of 0–3; (d) measurable disease; and (e) at least one response evaluation based on a computed tomography (CT) scan with a 5‐mm slice thickness completed after starting treatment with nivolumab. This study was approved by the Institutional Review Board of the National Cancer Center (approval number: 2017‐229). This research was conducted in accordance with the ethical principles set out in the Declaration of Helsinki.

### 2.2 Assessment

Patients were classified into SP or MP according to the definitions described above when target lesions and/or nontarget lesions exhibited progression and/or a new lesion appeared, as determined using the Response Evaluation Criteria in Solid Tumors (RECIST), version 1.1.^23^ Patients had CT scans every 6 weeks or when symptoms worsened. The progression pattern was evaluated by CT only, without positron emission tomography scans or scintigraphy. PFS was defined as the time from initiation of nivolumab treatment until progression or death or was censored in cases with survival without progression; OS was defined as the time from initiation of nivolumab treatment until death or was censored at the last follow‐up visit in surviving patients.

Overall tumor response was assessed and classified as a complete response (CR), a partial response (PR), stable disease (SD), or PD according to RECIST, version 1.1.^23^ As target lesions, up to five lesions in each patient and up to two lesions per organ were measured. The proportion of change in the sizes of the individual target lesion at the time of best response was measured and classified as CR (disappearance), PR (≤30% regression), SD (>30% regression and ≤20% progression), or PD (>20% progression). Accordingly, the objective response of an individual target lesion was defined as at least 30% regression.

Response rates were compared by the chi‐squared test. PFS, OS, and PPS were estimated using the Kaplan–Meier method and were compared using the log‐rank test. Prognostic factors for PFS and OS were explored using univariate and multivariate analyses with Cox regression models. Prognostic variables showing *p* < 0.10 in univariate analysis were considered potentially relevant and were included in the multivariate Cox regression analysis. The statistical analyses were performed using the SPSS software package (SPSS, Inc.), SAS version 9.4 (SAS Institute), and R version 4.0.2 software (R Core Team). All tests were two‐sided; *p* < 0.05 was considered statistically significant.

## 3 RESULTS

### 3.1 Patient characteristics

The subjects were 143 patients with advanced esophagogastric junction and gastric cancer who received nivolumab between March 2015 and May 2020 at the National Cancer Center Hospital. Sixty‐nine patients were excluded because of the absence of a target lesion (*n* = 35), no assessable CT examination (*n* = 19), prior use of an ICI as a first‐ or second‐line treatment (*n* = 9), or a histology other than adenocarcinoma (*n* = 6). Finally, 74 patients were identified as the subjects of this study. The baseline characteristics of the subjects are shown in Table A1. The median follow‐up period was 20.2 months (range, 6.7–70.6 months), and the median number of target lesions was two (range, 1–5). Forty‐six (62%) patients had lymph node metastasis (66 lesions), 37 (50%) patients had liver metastasis (68 lesions), 14 (19%) patients had peritoneal metastasis (22 lesions), and 11 (15%) patients had lung metastasis (16 lesions). There were 10 other target lesions in the pancreas, adrenal gland, and subcutaneous metastases.

### 3.2 Efficacy in all patients

The ORR was 8.1% (95% CI, 3.0%–16.8%), with one CR and five PRs, and the disease control rate was 39.2% (95% CI, 28.0%–51.2%). The median PFS was 1.8 months (95% CI, 1.5–2.4 months), and the 6‐, 12‐, 18‐, and 24‐month PFS rates were 12.5% (95% CI, 5.9%–21.7%), 8.9% (3.5%–17.6%), 8.9% (3.5%–17.6%), and 8.9% (3.5%–17.6%), respectively. The median OS was 6.8 months (95% CI, 4.2–10.0 months), and the 6‐, 12‐, 18‐ and 24‐month OS rates were 52.0% (95% CI, 39.4%–63.2%), 31.3% (19.9%–43.4%), 17.9% (8.2%–30.4%), and 14.9% (6.1%–27.3%), respectively (Figure A1,a,b).

### 3.3 Response at individual metastatic sites and change in the size of individual target lesions

**FIGURE 1:**
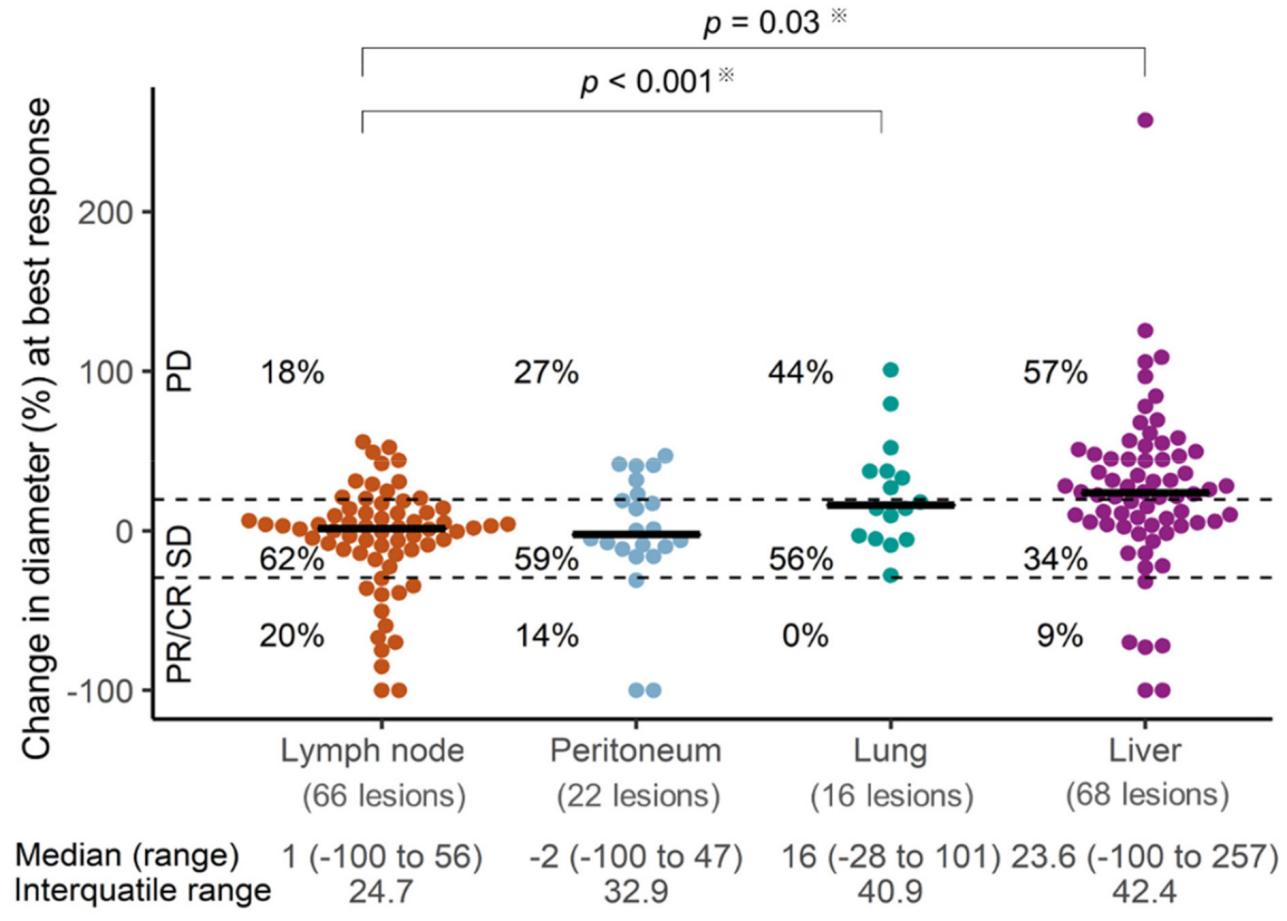
Distribution of changes in diameter at time of best response of individual target lesions according to metastatic site. CR, complete response; PR, partial response; SD, stable disease; PD, progression disease; ※PD rate.

The ORR according to metastatic site (lymph node, peritoneum, lung, and liver) were 20% (13/66), 14% (3/22), 0% (0/16), and 9% (6/68), respectively, while the PD rates were 18% (12/66), 27% (6/22), 44% (7/16), and 57% (39/68), respectively. The PD rate were significantly higher in the lung (*p* < 0.001) and the liver (*p* = 0.03) than in the lymph nodes (Figure 1).

**FIGURE 2:**
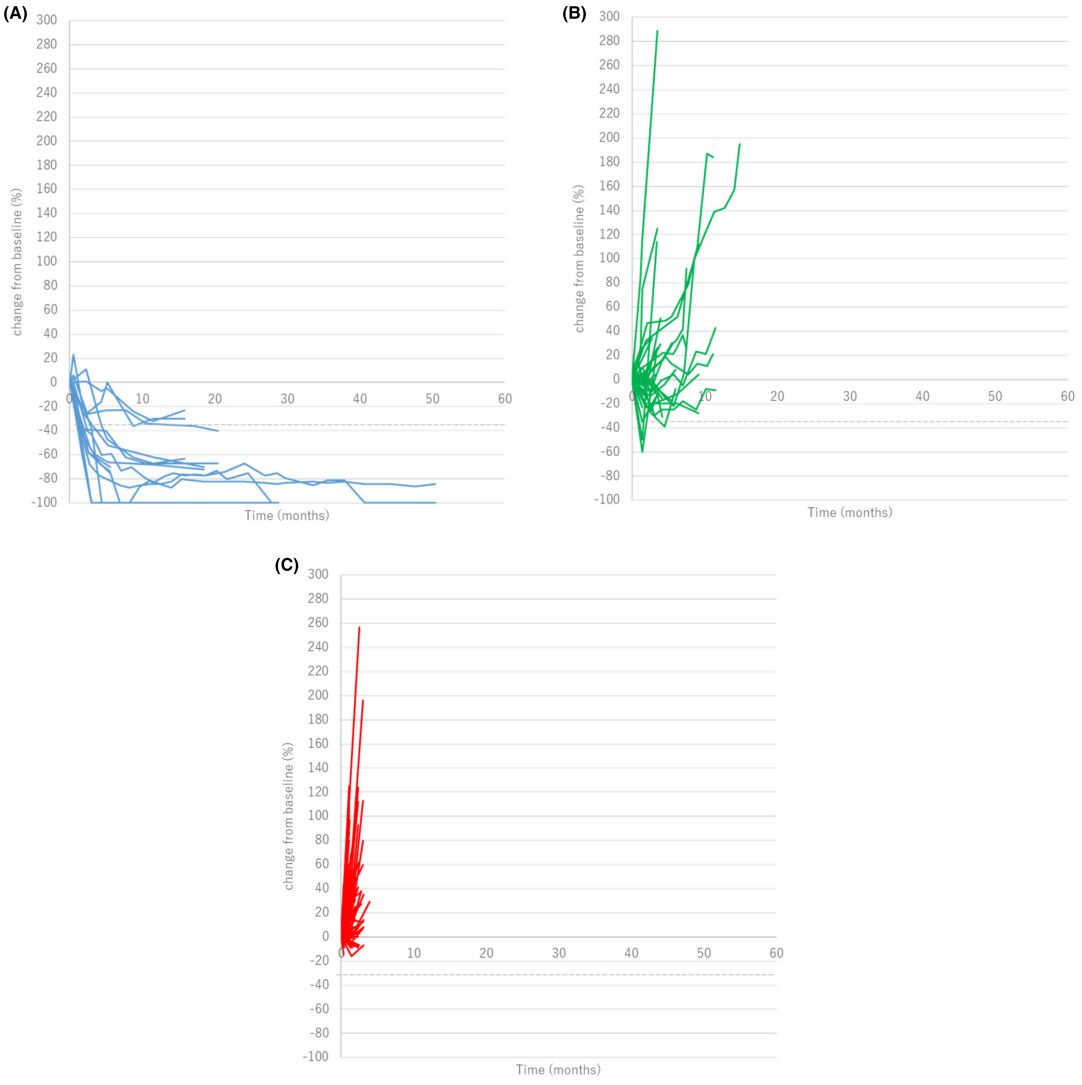
Spider plot showing the change in the size of individual target lesions. (A) Target lesions (*n* = 16) in patients with complete response (CR) or partial response (PR) as overall tumor response (*n* = 6). (B) Target lesions (*n* = 39) in patients with stable disease (SD) (*n* = 23). (C) Target lesions (*n* = 127) in patients with progression disease (PD) (*n* = 45).

The change in the sizes of individual target lesions were followed until nivolumab was discontinued (Figure 2A–C). In patients with CR or PR as overall tumor response, 16 of 16 (100%) of individual target lesions also achieved the objective response at the time of best response. On the other hand, in patients with SD or PD, the response of individual target lesions tended to be heterogeneous. There were 39 target lesions in patients with SD, and 30 lesions (76.9%) were SD, six lesions (15.4%) were PD, three lesions (7.7%) were PR. In patients with PD, 88 of 127 lesions (69.3%) were PD, while 39 lesions were SD (30.7%).

### 3.4 Patterns of progressions

**FIGURE 3:**
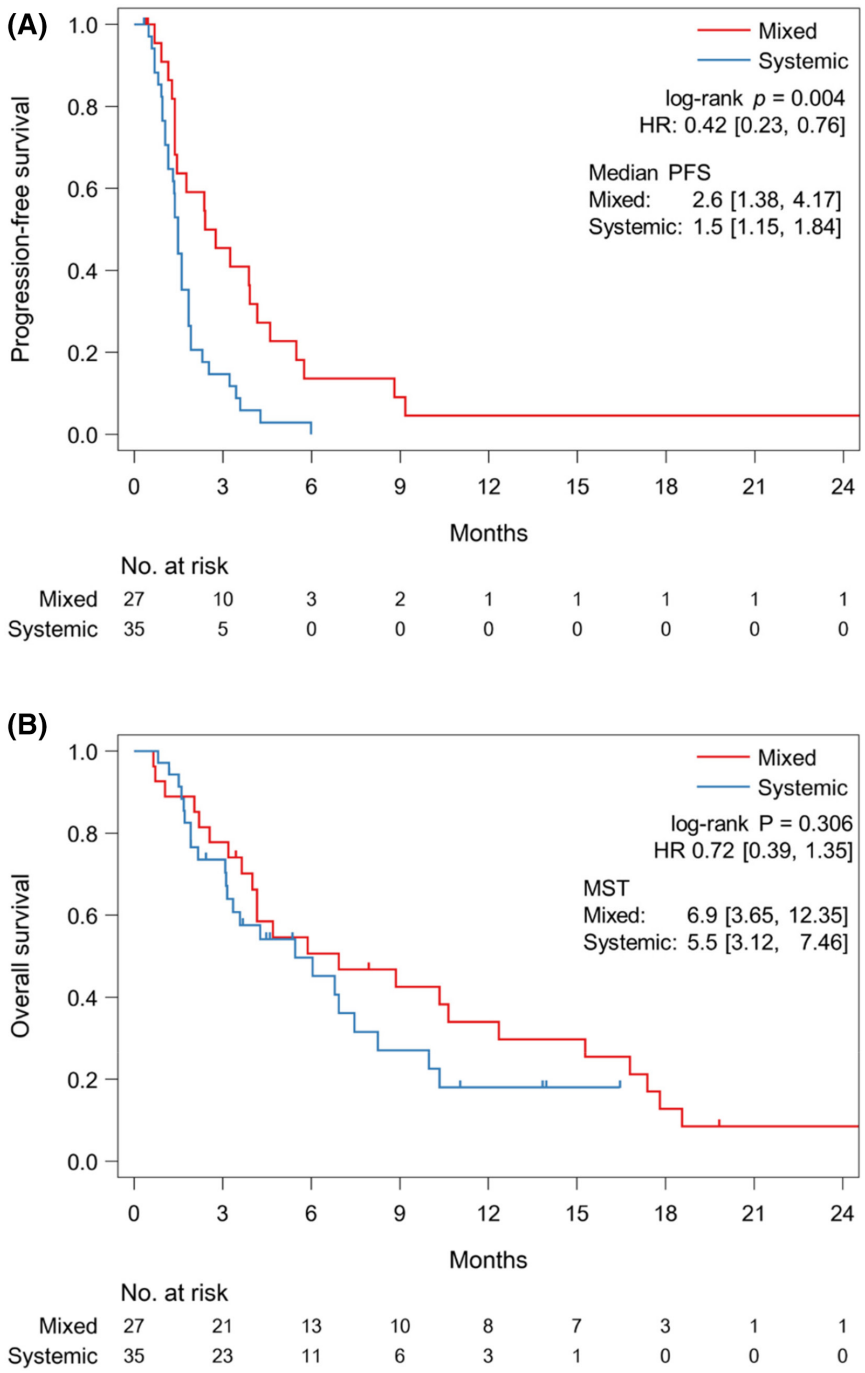
Kaplan–Meier curves showing (A) progression‐free survival (PFS) and (B) overall survival (OS) in patients with systemic progression and mixed progression.

Regarding the progression patterns, 35 (47%) and 27 (36%) patients had SP and MP, respectively, and 12 (16%) patients had no progression. Patient background of SP or MP are shown in Table 1. Patients with SP had higher incidences of HER2‐positivity and liver metastases and a lower incidence of peritoneal metastases. Patients with MP had a significantly longer PFS than those with SP (median, 2.6 vs. 1.5 months; HR, 0.42; 95% CI, 0.23–0.76; *p* = 0.004); however, the OS did not differ significantly between patients with MP and those with SP (median, 6.9 vs. 5.5 months; HR, 0.72; 95% CI, 0.39–1.35; *p* = 0.306) (Figure 3A,B).

**TABLE 1:** Characteristics of patients with systemic progression or mixed progression.

	Systemic (*n* = 35)	Mixed (*n* = 27)	*P*
Age, in years			
Median [range]	69 [36–87]	70 [33–87]	0.525
Sex			0.160
Male	28 (80%)	17 (63%)	
Female	7 (20%)	10 (37%)	
PS			0.747
0	7 (20%)	4 (15%)	
1	25 (71%)	22 (81%)	
2	2 (6%)	0 (0%)	
3	1 (3%)	1 (4%)	
Histology			0.215
Intestinal	16 (46%)	7 (26%)	
Diffuse	7 (20%)	10 (37%)	
Mixed	11 (31%)	10 (37%)	
Unknown	1 (3%)	0 (0%)	
HER2 status			0.009
Positive	14 (40%)	3 (11%)	
Negative	21 (60%)	22 (81%)	
Unknown	0 (0%)	2 (7%)	
Prior gastrectomy	14 (40%)	17 (63%)	0.124
Prior treatment			
Fluoropyrimidine	35 (100%)	27 (100%)	1
Platinum agents	31 (88.6%)	25 (92.6%)	0.689
Taxanes	32 (91.4%)	27 (100%)	0.250
Ramucirumab	18 (51.4%)	17 (63.0%)	0.442
Irinotecan	17 (48.6%)	14 (51.9%)	1
Trastuzumab	14 (40.0%)	3 (11.1%)	0.020
Number of prior regimens			0.502
2	16 (45.7%)	13 (48.1%)	
3	11 (31.4%)	6 (22.2%)	
≥4	8 (22.9%)	8 (29.6%)	
Dose of nivolumab			0.798
3 mg/kg	21 (60.0%)	15 (55.6%)	
240 mg/body	14 (40.0%)	12 (44.4%)	
Primary site			1
Gastric	31 (89%)	24 (89%)	
EGJ	4 (11%)	3 (11%)	
Site of metastases			
Lymph node	20 (57%)/29 lesions	20 (74%)/27 lesions	0.192
Liver	26 (74%)/48 lesions	7 (26%)/12 lesions	<0.001
Peritoneum	3 (9%)/4 lesions	9 (33%)/15 lesions	0.022
Lung	8 (23%)/12 lesions	3 (11%)/4 lesions	0.321

Abbreviations: EGJ, esophagogastric junction; PS, performance status.

**FIGURE 4:**
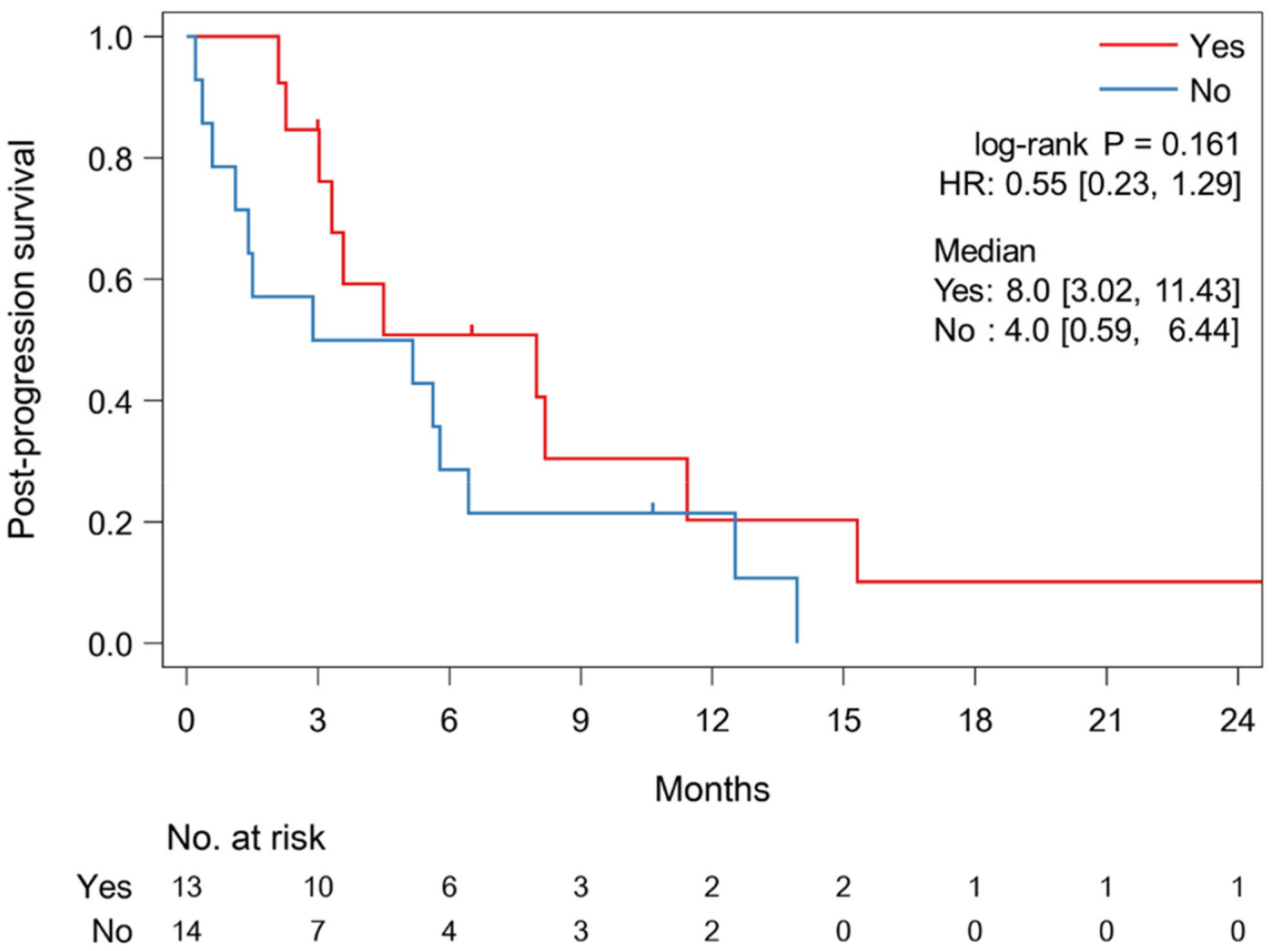
Kaplan–Meier curves showing post‐progression survival after initial mixed progression in 13 patients receiving treatment beyond mixed progression (TBP) with nivolumab (Yes) and 14 patients receiving no TBP (No).

In the MP group, 13 (48%) patients received TBP with nivolumab after initial MP. Although the histological type was unequally distributed between the TBP and no TBP groups (Table A2), other characteristics were generally balanced. The 13 patients with TBP tended to have a longer PPS than the 14 patients without TBP (median, 8.0 vs. 4.0 months; HR, 0.55; 95% CI, 0.23–1.29; *p* = 0.161) (Figure 4), and one patient (7.7%) experienced PR during TBP. The median time from MP to next PD during TBP was 1.4 months (95% CI, 0.9–1.9 months). By contrast, six (17%) patients received TBP with nivolumab after initial SP, and the backgrounds of the patients with or without TBP were similar in the SP group (Table A3). The six patients receiving TBP had a PPS comparable to that of the 29 patients not receiving TBP (median, 3.2 vs. 3.4 months; HR, 0.76; 95% CI, 0.28–2.05; *p* = 0.584) (Figure A2). No responses were seen during TBP.

### 3.5 Univariate and multivariate analyses

In the univariate analysis for PFS, no variables showed *p* < 0.10. Male had a trend of a longer OS (HR, 0.53; 95% CI, 0.27–1.02; *p* = 0.06) and a diffuse‐type histology (HR, 1.78; 95% CI, 0.95–3.35; *p* = 0.07), liver metastasis (HR, 1.73; 95% CI, 0.97–3.06; *p* = 0.06) and peritoneal metastasis (HR, 1.81; 95% CI, 0.99–3.31; *p* = 0.05) had a trend of a shorter OS. In the multivariate analysis, a diffuse‐type histology (HR, 2.24; 95% CI, 1.03–4.86; *p* = 0.04), liver metastasis (HR, 2.04; 95% CI, 1.15–3.65; *p* = 0.02) were associated with a shorter PFS. In addition, a diffuse‐type histology (HR, 2.36; 95% CI, 1.07–5.20; *p* = 0.03) and liver metastasis (HR, 2.52; 95% CI, 1.32–4.82; *p* < 0.01) were associated with a shorter OS (Table A4, A5).

## 4 DISCUSSION

This study revealed that the response of each target lesions was heterogeneous in patients with SD or PD and the response to nivolumab differed according to metastatic site in patients with advanced gastric cancer. Patients with MP had a significantly longer PFS than those with SP and they had the benefit of TBP with nivolumab.

Tumor heterogeneity in patients with advanced gastric cancer is well documented,^24^ and the intra‐tumoral heterogeneity of neoantigens is reportedly associated with a poor response to ICIs.^25^ Metastatic tumors originate from a subclone of the primary tumor, as shown by next‐generation sequencing.^26^ The present study showed that metastatic lesions in the lung and liver had a significantly poorer response to nivolumab than those in lymph nodes in patients with advanced gastric cancer. These results are consistent with a previous report that lymph node metastases achieved better responses to ICIs than lung and liver metastases in patients with NSCLC.^16^ Lymph nodes are important for generating and regulating immune responses to autoantigens and contain abundant CD8^+^ T‐cells.^27^ In addition, T‐cell priming takes place in lymph nodes where tumor‐specific antigens are drained. Tumor‐specific antigens expressed in tumor cells as a result of genetic and epigenetic alterations are presented by dendritic cells or antigen‐presenting cells to T‐cells; the activated T‐cells then attack the tumor cells.^27^, ^28^, ^29^, ^30^, ^31^ For this reason, lymph node metastases are responsive to ICIs. On the other hand, liver metastases have poor responses to ICIs, similar to the responses of other malignancies.^16^, ^18^, ^19^ The liver exhibits immunological tolerance because of its exposure to numerous antigens (not only microbes, but also food‐derived antigens arriving in the liver via the hepatic artery and portal vein).^32^ Several mechanisms including the trapping and isolation of activated CD8^+^ T‐cells,^33^ clonal anergy,^34^, ^35^ clonal preference for Th2 cells,^36^, ^37^ and T‐cell exhaustion^38^ could be mechanisms for immune resistance in the liver. A recent study showed significantly fewer tumor‐infiltrating CD8^+^ T‐cells in non‐hepatic metastases in patients with liver metastasis, in comparison with the situation in patients without liver metastasis.^17^ In a mouse model, liver metastases led to the systemic depletion of T‐cells that recognize antigen‐specific.^39^ Thus, in addition to liver metastases having a poor response to ICIs, they are also associated with a poor response systemically. In this study, liver metastasis was associated with shorter PFS and OS in a multivariate analysis. Similarly, the lung is exposed to numerous environmental antigens via the airway, leading to immune tolerance by regulatory T‐cells.^40^, ^41^ For this reason, lesions in the lung and liver respond poorly to ICIs. Additional studies are needed to clarify the mechanisms that are involved.

Since the response to ICIs differs according to metastatic site, the progression pattern is also heterogeneous. In this study, we confirmed that target lesions in advanced gastric cancer patients with PR or CR were reduced uniformly, while those in patients with SD or PD responded heterogeneously. Then, the progression patterns were classified into SP and MP based on a previous report investigating the prognostic impact of progression pattern.^16^ In advanced gastric cancer, PFS was significantly longer for patients with MP compared to patients with SP, with an HR of 0.42. Although the OS did not significantly differ between patients with MP and those with SP, median survival in those with MP was 1.4 months longer than those with SP, with an HR of 0.72. In addition, patients with TBP with nivolumab tended to have a longer PPS than those without TBP in patients with MP, and one patient experienced tumor regression after MP. Because few effective chemotherapies are available as later‐line treatments for patients with advanced gastric cancer, the continuation of nivolumab after MP might be an option if the patient is in good physical condition.^13^ On the other hand, TBP after SP is not recommended as it was ineffective. Among patients with MP, patients who could receive nivolumab TBP did not have diffuse‐type histology. In this study, multivariate analysis has shown diffuse‐type histology as a poor prognostic factor. Patients with diffuse‐type histology has a poor prognosis, which may make TBP with nivolumab difficult. However, this study is retrospective and small, and further research is needed on the optimal patients for TBP with nivolumab.

HER2‐positive advanced gastric cancer showed more SP in this study. This might be because HER2 positivity is significantly associated with liver metastasis.^42^ In this study, more patients in the SP group were HER2‐positive and had liver metastases. Liver metastasis responded poorly to nivolumab. The relation between HER2 status and the efficacy of ICIs has been still controversial. The exploratory analysis in the ATTRACTION‐2 trial adopted prior trastuzumab use as surrogate for HER2 positive. It showed a trend of a longer OS in patients with prior trastuzumab use than those without prior trastuzumab use (median, 8.3 months vs. 4.8 months).^43^ The KEYNOTE‐811 trial evaluating the combination of anti‐PD‐1 antibody and HER2‐blockade plus chemotherapy showed remarkable ORR of 74.4%.^44^ Meanwhile, it has been reported that the tumor microenvironment of HER2‐positive gastric cancer has low expression of cytotoxic T‐lymphocyte antigen 4 (CTLA‐4), lymphocyte activation gene 3 (LAG‐3), T‐cell immunoreceptor with immunoglobulin and ITIM domains (TIGIT), PD‐1 and PD‐L1.^42^ It suggests that ICIs may be less effective against HER2‐positive gastric cancer.

This study had some limitations. First, this was a retrospective study by a single institution. Second, we did not assess Epstein–Barr virus (EBV) infection, microsatellite instability (MSI), and PD‐L1 expression. It has been suggested that EBV infection status in gastric cancer may be a biomarker for ICIs, but this remains unclear.^45^ PD‐L1 expression has been shown to be a predictor of ICI efficacy in many studies,^46^, ^47^, ^48^ however its impact in patients with advanced gastric cancer treated with nivolumab remains controversial. In the ATTRACTION‐2 trial, the median OS was not different between the PD‐L1‐positive group and the PD‐L1‐negative group.^10^ The PD‐L1 positivity in the study was assessed by tumor proportional score, however an exploratory analysis showed that high combined positivity score was associated with good prognosis.^49^ Further investigation of optimal biomarkers for ICIs is needed. Third, the timing of CT evaluation may differ between the SP and MP group because tumor growth leads to worsening of clinical symptoms especially in SP group. It may have affected PFS. Forth, this study included patients who had measurable target lesions. In case of peritoneal metastasis, patients with massive ascites without measurable target lesions were excluded and it could cause selection bias. However, those patients are often in poor general condition, and the indication for chemotherapy should be carefully considered.

In conclusion, the response to nivolumab varied according to metastatic site, resulting in different progression patterns, and PFS was significantly longer for patients with MP than those with SP. The continuation of nivolumab treatment after MP might be a useful treatment option. Future studies are warranted to investigate the biological mechanisms of the different responses according to metastatic site and biomarkers for selecting candidates for treatment with ICIs beyond progression.

## AUTHOR CONTRIBUTIONS

**Toru Kadono:** Conceptualization (lead); data curation (lead); formal analysis (equal); writing – original draft (lead). **Satoru Iwasa:** Conceptualization (equal); data curation (equal); formal analysis (equal); validation (equal); writing – review and editing (equal). **Kengo Nagashima:** Conceptualization (equal); formal analysis (lead); validation (equal); writing – review and editing (equal). **Kotoe Oshima:** Conceptualization (equal); validation (equal); writing – review and editing (equal). **Shun Yamamoto:** Conceptualization (equal); validation (equal); writing – review and editing (equal). **Hedekazu Hirano:** Conceptualization (equal); validation (equal); writing – review and editing (equal). **Natsuko Okita:** Conceptualization (equal); validation (equal); writing – review and editing (equal). **Hirokazu Shoji:** Conceptualization (equal); validation (equal); writing – review and editing (equal). **Yoshitaka Honma:** Conceptualization (equal); validation (equal); writing – review and editing (equal). **Atsuo Takashima:** Conceptualization (equal); validation (equal); writing – review and editing (equal). **Ken Kato:** Conceptualization (equal); validation (equal); writing – review and editing (equal). **Toshikazu Ushijima:** Conceptualization (equal); validation (equal); writing – review and editing (equal). **Narikazu Boku:** Conceptualization (equal); validation (equal); writing – review and editing (equal).

## FUNDING INFORMATION

This study received no specific grant from any backing agency in the public, marketable, or not‐for‐profit sectors.

## CONFLICT OF INTEREST STATEMENT

Author SI has received research grants and a speaker honorarium from Ono Pharmaceutical and Bristol‐Myers Squibb. Author KN has received consulting fees from Toray Industries, Inc. and a speaker honorarium from Pfizer R&D Japan G.K. Author SY has received research grants and a speaker honorarium from Ono Pharmaceutical and Bristol‐Myers Squibb. Author HH has received research grants and a speaker honorarium from Ono Pharmaceutical and Bristol‐Myers Squibb. Author NO has received research grants from Ono Pharmaceutical and Bristol‐Myers Squibb. Author HS has received research grants and a speaker honorarium from Ono Pharmaceutical and Bristol‐Myers Squibb. Author YH has received research grants and a speaker honorarium from Ono Pharmaceutical and Bristol‐Myers Squibb. Author AT has received research grants and a speaker honorarium from Ono Pharmaceutical and Bristol‐Myers Squibb. Author KK has received research grants and a speaker honorarium from Ono Pharmaceutical and Bristol‐Myers Squibb. Author TU has received research grants and a speaker honorarium from Company Ono Pharmaceutical and Bristol‐Myers Squibb. Author NB has received research grants from Ono Pharmaceutical and Takeda Pharmaceutical and a speaker honorarium from Ono Pharmaceutical, Bristol‐Myers Squibb, Taiho Pharmaceutical and Daiichi‐Sankyo. The other authors declare that they have no conflict of interest.

## ETHICAL APPROVAL

This study was approved by the Institutional Review Board of the National Cancer Center, Japan (approval number: 2017‐229). This study was conducted in accordance with the ethical principles outlined in the Declaration of Helsinki.

## PATIENT CONSENT

Patient consent was obtained through an opt‐out method.

## Supporting information


Appendix.

## Data Availability

All data generated or analyzed during this study are included in this published article (and its Supplementary Information files).
