# Supplementary material for: Progression patterns and site‐specific responses in advanced gastric cancer patients treated with nivolumab
Source: Cancer Med. 2023 Feb 15;12(8):9322–31. doi: 10.1002/cam4.5689 (PMC10166927; doi:10.1002/cam4.5689)

**Supplementary Information**

Table A1. Characteristics of all patients with advanced gastric cancer treated with nivolumab

|  | No. of patients (n = 74) |  |
| --- | --- | --- |
| Age, in years |  |  |
| Median [range] | 69 [33-87] |  |
| Sex |  |  |
| Male/Female | 55 (74%)/19 (26%) |  |
| PS |  |  |
| 0/1/2/3 | 15 (20%)/53 (72%)/4 (5%)/2 (3%) |  |
| Histology |  |  |
| intestinal/diffuse/mixed/unknown | 30 (41%)/20 (27%)/22 (30%)/2 (3%) |  |
| HER2 status |  |  |
| positive/negative/unknown | 19 (26%)/53 (72%)/2 (3%) |  |
| Prior gastrectomy | 37 (50%) |  |
| Prior treatment |  |  |
| Fluoropyrimidine | 74 (100%) |  |
| Platinum agents | 66 (89%) |  |
| Taxanes | 71 (96%) |  |
| Ramucirumab | 43 (58%) |  |
| Irinotecan | 35 (47%) |  |
| Trastuzumab | 19 (26%) |  |
| Number of prior regimens |  |  |
| 2 | 35 (47%) |  |
| 3 | 21 (28%) |  |
| ≥4 | 18 (24%) |  |
| Dose of Nivolumab |  |  |
| 3 mg/kg | 40 (54%) |  |
| 240 mg/body | 34 (46%) |  |
| Primary site |  |  |
| Gastric/EGJ | 66 (89%)/8 (11%) |  |
| Site of metastases |  |  |
| Lymph node | 46 (62%)/66 lesions |  |
| Liver | 37 (50%)/68 lesions |  |
| Peritoneum | 14 (19%)/22 lesions |  |
| Lung | 11 (15%)/16 lesions |  |
| Others | 8(10%)/9 lesions |  |

PS, performance status; EGJ, esophagogastric junction

Table A2. Twenty-seven patients with mixed progression who had received treatment beyond progression (TBP) with nivolumab or who had not received TBP (no TBP)

|  | TBP (n = 13) | no TBP (n = 14) | *P* |
| --- | --- | --- | --- |
| Age, in years |  |  |  |
| Median [range] | 68 [61-79] | 71.5 [33-87] | 0.753 |
| Sex |  |  | 0.236 |
| Male | 10 (77%) | 7 (50%) |  |
| Female | 3 (23%) | 7 (50%) |  |
| PS |  |  | 0.455 |
| 0 | 1 (8%) | 3 (21%) |  |
| 1 | 12 (92%) | 10 (71%) |  |
| 2 | 0 (0%) | 0 (0%) |  |
| 3 | 0 (0%) | 1 (7%) |  |
| Histology |  |  | < 0.001 |
| Intestinal | 7 (54%) | 0 (0%) |  |
| Diffuse | 0 (0%) | 10 (71%) |  |
| Mixed | 6 (46%) | 4 (29%) |  |
| Unknown | 0 (0%) | 0 (0%) |  |
| HER2 status |  |  | 0.789 |
| Positive | 10 (77%) | 12 (86%) |  |
| Negative | 2 (15%) | 1 (7%) |  |
| Unknown | 1 (8%) | 1 (7%) |  |
| Prior gastrectomy | 10 (77%) | 7 (50%) | 0.222 |
| Prior treatment |  |  |  |
| Fluoropyrimidine | 13 (100%) | 14 (100%) | 1 |
| Platinum agents | 12 (92%) | 13 (93%) | 1 |
| Taxanes | 13 (100%) | 14 (100%) | 1 |
| Ramucirumab | 6 (46%) | 11 (79%) | 0.120 |
| Irinotecan | 8 (62%) | 6 (43%) | 0.449 |
| Trastuzumab | 2 (15%) | 1 (7%) | 0.596 |
| Number of prior regimens |  |  | 0.612 |
| 2 | 5 (39%) | 8 (57%) |  |
| 3 | 3 (23%) | 3 (21%) |  |
| ≥4 | 5 (39%) | 3 (21%) |  |
| Dose of Nivolumab |  |  | 0.252 |
| 3 mg/kg | 9 (69%) | 6 (43%) |  |
| 240 mg/body | 4 (31%) | 8 (57%) |  |
| Primary site |  |  | 0.236 |
| Gastric | 13 (100%) | 11 (79%) |  |
| EGJ | 0 (0%) | 3 (21%) |  |
| Site of metastases |  |  |  |
| Lymph node | 10 (77%) | 10 (71%) | 1 |
| Liver | 5 (38%) | 2 (14%) | 0.209 |
| Peritoneum | 6 (46%) | 3 (21%) | 0.236 |
| Lung | 1 (8%) | 2 (14%) | 1 |
| Time to mixed progression, months |  |  | 0.500 |
| Median [range] | 1.38 [0.43-8.80] | 1.53 [0.36-9.17] |  |
| Best response before mixed progression |  |  | 0.704 |
| SD | 8 (62%) | 7 (50%) |  |
| PD | 5 (38%) | 7 (50%) |  |

TBP, treatment beyond progression; PS, performance status; EGJ, esophagogastric junction

Table A3. Thirty-five patients with systemic progression who had received treatment beyond progression (TBP) with nivolumab or who had not received TBP (no TBP)

|  | TBP (n = 6) | no TBP (n = 29) | *P* |
| --- | --- | --- | --- |
| Age, in years |  |  |  |
| Median [range] | 59.5 [45-81] | 69 [36-87] | 0.268 |
| Sex |  |  | 0.576 |
| Male | 4 (67%) | 24 (83%) |  |
| Female | 2 (33%) | 5 (17%) |  |
| PS |  |  | 0.771 |
| 0 | 2 (33%) | 5 (17%) |  |
| 1 | 4 (67%) | 21 (72%) |  |
| 2 | 0 (0%) | 2 (7%) |  |
| 3 | 0 (0%) | 1 (3%) |  |
| Histology |  |  | 0.610 |
| Intestinal | 3 (50%) | 13 (45%) |  |
| Diffuse | 2 (33%) | 5 (17%) |  |
| Mixed | 1 (17%) | 10 (34%) |  |
| Unknown | 0 (0%) | 1 (3%) |  |
| HER2 status |  |  | 0.366 |
| Positive | 5 (83%) | 16 (55%) |  |
| Negative | 1 (17%) | 13 (45%) |  |
| Unknown | 0 (0%) | 0 (0%) |  |
| Prior gastrectomy | 4 (67%) | 10 (34%) | 0.191 |
| Prior treatment |  |  |  |
| Fluoropyrimidine | 6 (100%) | 29 (100%) | 1 |
| Platinum agents | 5 (83%) | 26 (90%) | 0.546 |
| Taxanes | 5 (83%) | 27 (93%) | 0.442 |
| Ramucirumab | 1 (17%) | 17 (59%) | 0.088 |
| Irinotecan | 4 (67%) | 13 (45%) | 0.402 |
| Trastuzumab | 1 (17%) | 13 (45%) | 0.366 |
| Number of prior regimens |  |  | 0.781 |
| 2 | 2 (33%) | 14 (48%) |  |
| 3 | 2 (33%) | 9 (31%) |  |
| ≥4 | 2 (33%) | 6 (21%) |  |
| Dose of Nivolumab |  |  | 1 |
| 3 mg/kg | 4 (67%) | 17 (59%) |  |
| 240 mg/body | 2 (33%) | 12 (41%) |  |
| Primary site |  |  | 0.546 |
| Gastric | 5 (83%) | 26 (90%) |  |
| EGJ | 1 (17%) | 3 (10%) |  |
| Site of metastases |  |  |  |
| Lymph node | 3 (50%) | 17 (59%) | 1 |
| Liver | 4 (67%) | 22 (76%) | 0.636 |
| Peritoneum | 1 (17%) | 2 (7%) | 0.442 |
| Lung | 3 (50%) | 5 (17%) | 0.117 |
| Time to systemic progression, months |  |  | 0.375 |
| Median [range] | 1.48 [0.33-4.27] | 1.48 [0.49-5.98] |  |
| Best response before systemic progression |  |  | 0.465 |
| PR | 0 (0%) | 2 (7%) |  |
| SD | 2 (33%) | 3 (10%) |  |
| PD | 4 (67%) | 24 (83%) |  |

TBP, treatment beyond progression; PS, performance status; EGJ, esophagogastric junction

Table A4. Univariate and multivariate analyses for PFS with Cox regression models. Four patients were excluded because of a lack of histology data.

| Covariate | Univariate analysis (n = 70) | | | | | Multivariate analysis (n = 70) | | | | |
| --- | --- | --- | --- | --- | --- | --- | --- | --- | --- | --- |
|  | HR | | 95% CI | | *P* value | HR | 95% CI | | | *P* value |
| Age |  |  | |  |  |  |  |  |  | |
| < 69 years | Reference |  | |  |  |  |  |  |  | |
| ≥ 69 years | 0.97 | 0.57 | | 1.64 | 0.90 |  |  |  |  | |
| Sex |  |  | |  |  |  |  |  |  | |
| Female | Reference |  | |  |  | Reference |  |  |  | |
| Male | 0.65 | 0.35 | | 1.23 | 0.19 | 0.80 | 0.41 | 1.58 | 0.52 | |
| PS |  |  | |  |  |  |  |  |  | |
| 0 | Reference |  | |  |  |  |  |  |  | |
| 1-3 | 1.46 | 0.75 | | 2.83 | 0.26 |  |  |  |  | |
| Histology |  |  | |  |  |  |  |  |  | |
| Intestinal | Reference |  | |  |  | Reference |  |  |  | |
| Diffuse | 1.51 | 0.82 | | 2.79 | 0.18 | 2.24 | 1.03 | 4.86 | 0.04 | |
| Mixed | 1.25 | 0.73 | | 2.15 | 0.42 | 1.70 | 0.85 | 3.37 | 0.13 | |
| HER2 status |  |  | |  |  |  |  |  |  | |
| Positive | Reference |  | |  |  |  |  |  |  | |
| Negative | 1.02 | 0.57 | | 1.83 | 0.93 |  |  |  |  | |
| Prior gastrectomy |  |  | |  |  |  |  |  |  | |
| No | Reference |  | |  |  |  |  |  |  | |
| Yes | 1.00 | 0.59 | | 1.70 | 0.99 |  |  |  |  | |
| Prior Ramucirumab use |  |  | |  |  |  |  |  |  | |
| No | Reference |  | |  |  |  |  |  |  | |
| Yes | 1.15 | 0.68 | | 1.96 | 0.60 |  |  |  |  | |
| Number of prior regimens |  |  | |  |  |  |  |  |  | |
| 2 | Reference |  | |  |  |  |  |  |  | |
| ≥ 3 | 1.12 | 0.66 | | 1.91 | 0.68 |  |  |  |  | |
| Primary site |  |  | |  |  |  |  |  |  | |
| Gastric | Reference |  | |  |  |  |  |  |  | |
| EGJ | 1.29 | 0.55 | | 3.06 | 0.56 |  |  |  |  | |
| Lymph node metastasis |  |  | |  |  |  |  |  |  | |
| No | Reference |  | |  |  |  |  |  |  | |
| Yes | 0.79 | 0.42 | | 1.48 | 0.46 |  |  |  |  | |
| Liver metastasis | |  | |  |  |  |  |  |  | |
| No | Reference |  | |  |  | Reference |  |  |  | |
| Yes | 1.55 | 0.91 | | 2.64 | 0.11 | 2.04 | 1.15 | 3.65 | 0.02 | |
| Peritoneum metastasis |  |  | |  |  |  |  |  |  | |
| No | Reference |  | |  |  | Reference |  |  |  | |
| Yes | 1.23 | 0.72 | | 2.11 | 0.45 | 1.20 | 0.68 | 2.13 | 0.53 | |
| Lung metastasis |  |  | |  |  |  |  |  |  | |
| No | Reference |  | |  |  |  |  |  |  | |
| Yes | 1.20 | 0.68 | | 2.14 | 0.53 |  |  |  |  | |

PS, Performance Status; EGJ, esophagogastric junction

Table A5. Univariate and multivariate analyses for OS with Cox regression models. Four patients were excluded because of a lack of histology data.

| Covariate | Univariate analysis (n = 70) | | | | | Multivariate analysis (n = 70) | | | | |
| --- | --- | --- | --- | --- | --- | --- | --- | --- | --- | --- |
|  | HR | | 95% CI | | *P* value | HR | 95% CI | | | *P* value |
| Age |  |  | |  |  |  |  |  |  | |
| < 69 years | Reference |  | |  |  |  |  |  |  | |
| ≥ 69 years | 0.63 | 0.36 | | 1.12 | 0.11 |  |  |  |  | |
| Sex |  |  | |  |  |  |  |  |  | |
| Female | Reference |  | |  |  | Reference |  |  |  | |
| Male | 0.53 | 0.27 | | 1.02 | 0.06 | 0.52 | 0.25 | 1.06 | 0.07 | |
| PS |  |  | |  |  |  |  |  |  | |
| 0 | Reference |  | |  |  |  |  |  |  | |
| 1-3 | 1.59 | 0.74 | | 3.41 | 0.23 |  |  |  |  | |
| Histology |  |  | |  |  |  |  |  |  | |
| Intestinal | Reference |  | |  |  | Reference |  |  |  | |
| Diffuse | 1.78 | 0.95 | | 3.35 | 0.07 | 2.36 | 1.07 | 5.20 | 0.03 | |
| Mixed | 0.97 | 0.53 | | 1.79 | 0.92 | 1.35 | 0.64 | 2.87 | 0.43 | |
| HER2 status |  |  | |  |  |  |  |  |  | |
| Positive | Reference |  | |  |  |  |  |  |  | |
| Negative | 0.83 | 0.43 | | 1.59 | 0.57 |  |  |  |  | |
| Prior gastrectomy |  |  | |  |  |  |  |  |  | |
| No | Reference |  | |  |  |  |  |  |  | |
| Yes | 0.80 | 0.45 | | 1.41 | 0.44 |  |  |  |  | |
| Prior Ramucirumab use |  |  | |  |  |  |  |  |  | |
| No | Reference |  | |  |  |  |  |  |  | |
| Yes | 0.91 | 0.51 | | 1.60 | 0.74 |  |  |  |  | |
| Number of prior regimens |  |  | |  |  |  |  |  |  | |
| 2 | Reference |  | |  |  |  |  |  |  | |
| ≥ 3 | 1.16 | 0.65 | | 2.07 | 0.63 |  |  |  |  | |
| Primary site |  |  | |  |  |  |  |  |  | |
| Gastric | Reference |  | |  |  |  |  |  |  | |
| EGJ | 1.00 | 0.42 | | 2.35 | 0.99 |  |  |  |  | |
| Lymph node metastasis |  |  | |  |  |  |  |  |  | |
| No | Reference |  | |  |  |  |  |  |  | |
| Yes | 0.88 | 0.45 | | 1.74 | 0.72 |  |  |  |  | |
| Liver metastasis | |  | |  |  |  |  |  |  | |
| No | Reference |  | |  |  | Reference |  |  |  | |
| Yes | 1.73 | 0.97 | | 3.06 | 0.06 | 2.52 | 1.32 | 4.82 | < 0.01 | |
| Peritoneum metastasis |  |  | |  |  |  |  |  |  | |
| No | Reference |  | |  |  | Reference |  |  |  | |
| Yes | 1.81 | 0.99 | | 3.31 | 0.05 | 1.81 | 0.98 | 3.33 | 0.06 | |
| Lung metastasis |  |  | |  |  |  |  |  |  | |
| No | Reference |  | |  |  |  |  |  |  | |
| Yes | 0.91 | 0.48 | | 1.76 | 0.79 |  |  |  |  | |

PS, Performance Status; EGJ, esophagogastric junction

**Figure Captions**

**Fig. A1**

(a) Kaplan-Meier curves showing progression-free survival (PFS) for all the patients. The PFS rates at 6, 12, 18 and 24 months were 12.5% (95% CI, 5.9%-21.7%), 8.9% (3.5%-17.6%), 8.9% (3.5%-17.6%) and 8.9% (3.5%-17.6%), respectively

(b) Kaplan-Meier curves showing overall survival (OS) for all the patients. The OS rates at 6, 12, 18 and 24 months were 52.0% (95% CI, 39.4%-63.2%), 31.3% (19.9%-43.4%), 17.9% (8.2%-30.4%) and 14.9% (6.1%-27.3%) respectively

**Fig. A2**

Kaplan-Meier curves showing post-progression survival after first systemic progression in 6 patients who received treatment beyond progression (TBP) with nivolumab (NO) and 29 patients who received no TBP (YES)

Figure A1. (a)


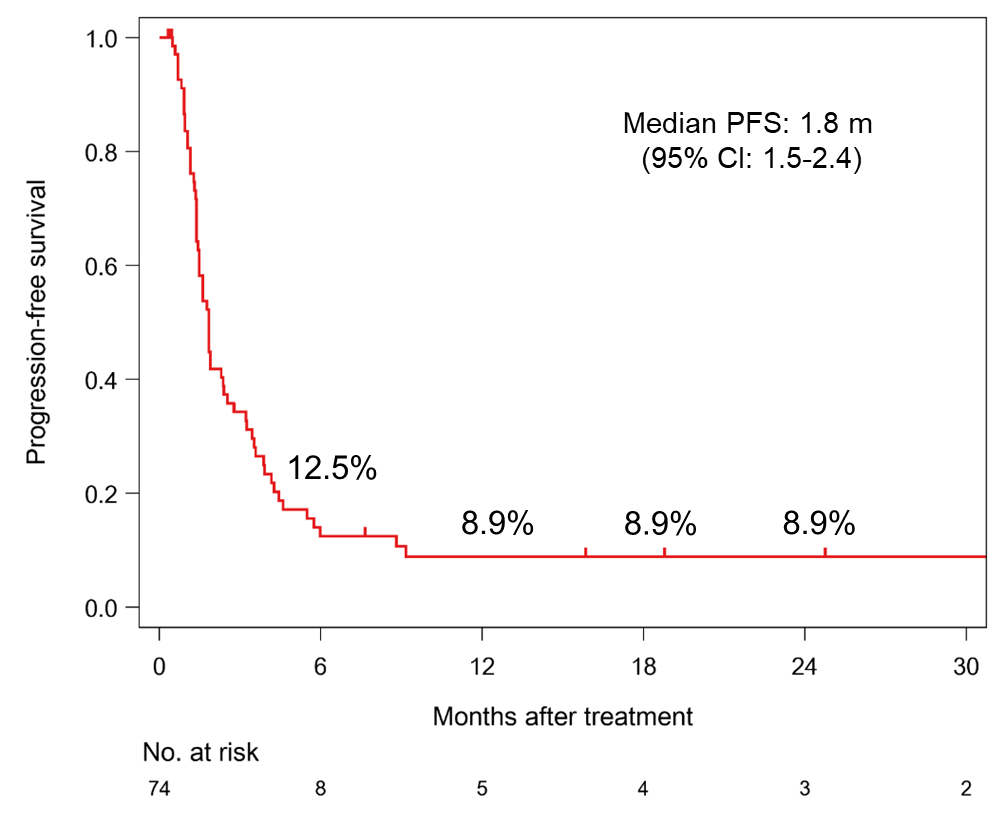


(b)


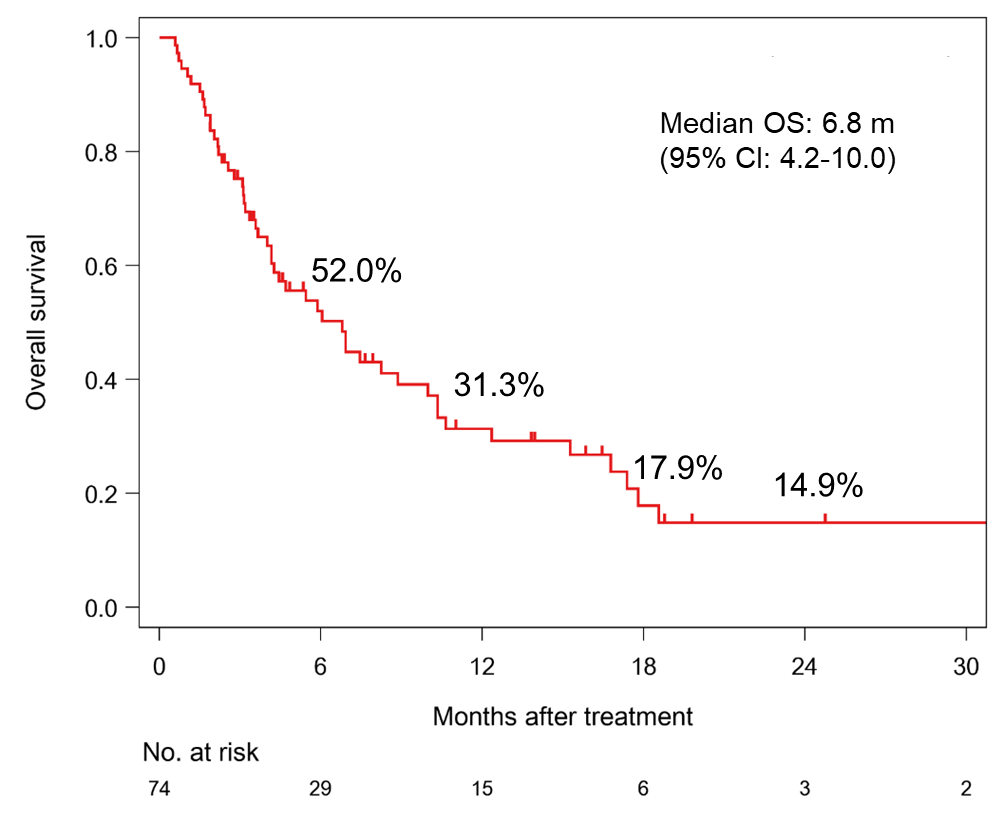


Figure A2.


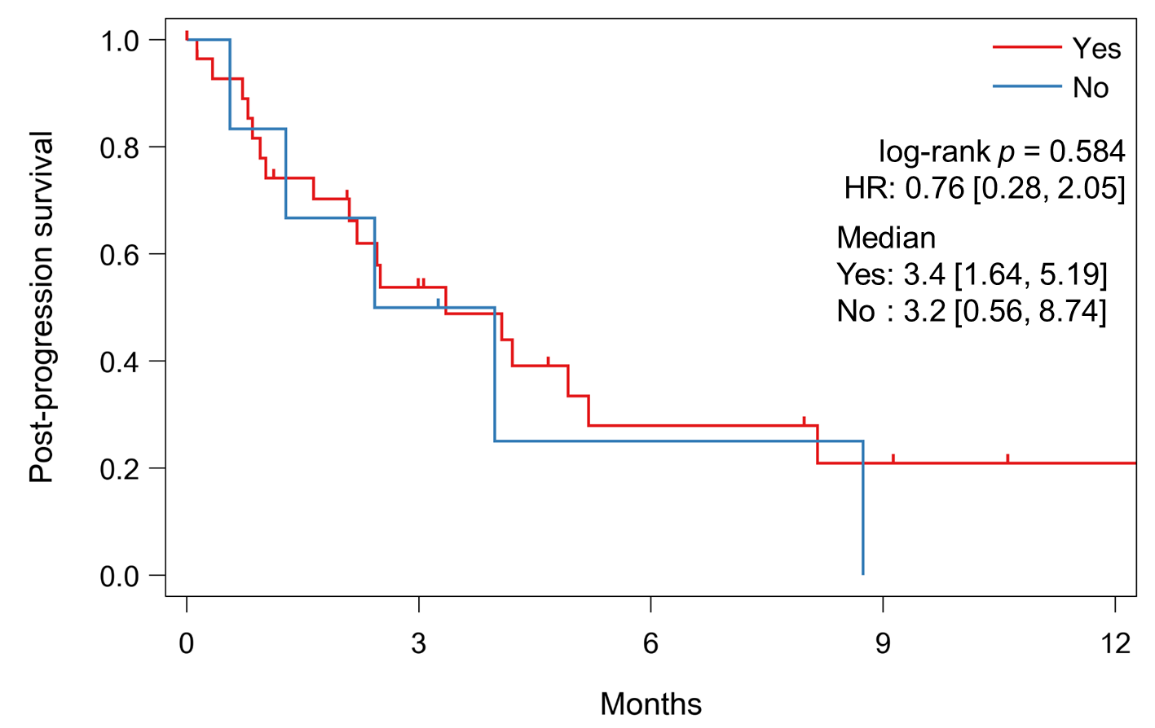

Supplement: Supplementary file 1 — Appendix. [file CAM4-12-9322-s001.docx]
